# Effectively training neural networks for stock index prediction: Predicting the S&P 500 index without using its index data

**DOI:** 10.1371/journal.pone.0230635

**Published:** 2020-04-10

**Authors:** Jinho Lee, Jaewoo Kang

**Affiliations:** Department of Computer Science and Engineering, Korea University, Seoul, Korea; The Bucharest University of Economic Studies, ROMANIA

## Abstract

We propose a novel method for training neural networks to predict the future prices of stock indexes. Unlike previous works, we do not use target stock index data for training neural networks for index prediction. Instead, we use only the data of individual companies to obtain sufficient amount of data for training neural networks for stock index prediction. As a result, our method can avoid various problems due to training complex machine learning models on a small amount of data. We performed numerous types of experiments to test methods designed for predicting the future price of the S&P 500 which is one of the most commonly traded stock indexes. Our experiments show that neural networks trained using our method outperform neural networks trained on stock index data. Compared with other state-of-the-art methods, our method is conceptually simpler and easier to apply, and achieves better results. We obtained approximately a 5-16% annual return before transaction costs during the test period (2006-2018).

## Introduction

Predicting future prices of stock indexes such as the S&P 500 or the Nasdaq Composite is a challenging and important task. A stock index is a collection of equities or securities usually computed from the market capital of constituents. Therefore, a stock index provides a summary of overall market performance and helps investors in their investing activities. Also, increasing the number of stock index-related securities has diversified the investing strategies of investors. An investor can predict the future prices of stock indexes to hedge against market risk or look for profitable opportunities. For these reasons, many previous works not only in finance but also in computer science have focused on predicting prices of stock indexes.

With the success of Neural Networks (NNs), especially in the computer science domain, several recent works have adopted NNs for stock market prediction. Some review papers [1, 2] show that NNs are one of the most commonly used machine learning techniques for predicting the future prices of individual stocks and stock indexes. Previous works used various types of data as input features, which include macro economic indicators [3], investors’ sentiment in web services [4, 5], search query frequency [6], or the information extracted from financial news articles [7, 8]. However, for stock index prediction, the majority of the recent works used historical price and volume data [9, 10] as input features because such data are publicly free and easy to obtain.

Regardless of the domain, training NNs on a sufficient amount of training data is crucial. For example, Sun et al. [12] showed that the performance of NN-based image classification models increased logarithmically with the size of the training data. Also, training complex machine learning models on a small amount of data often leads to overfitting. Although there is enough data for training NNs to predict the future prices of individual stocks, there is an insufficient amount of data for training NNs to predict future stock index prices. Because unlike individual stocks, the number of stock indexes is very small. If a NN is trained to predict the future price of the S&P 500 and a daily scale is used, only about 250 data points can be obtained per year.

To address this problem, many previous works lengthened the training period. For example, data collected over 10 years was used instead of data collected over only 1 year for training. In fact, most of the previous works used data collected over a long period for training their models to predict the future prices of stock indexes. However, increasing the size of the training data by simply collecting data over longer periods of time is a limited approach. For example, collecting data that spans periods of 50 or 100 years is infeasible. Moreover, NNs trained on relatively old data could be less effective in predicting the future prices of stock indexes. Also, we argue that only several thousands of data points are an insufficient amount of data for effectively training complex NNs to predict the future prices of stock indexes. In various fields, such as natural language processing [11] or image classification [12], many works have recently shown that training NNs on a larger amount of training data increases the performance of models.

The most critical problem is using stock index data for training NNs to predict the future price of a stock index. For example, lets assume that S&P 500 daily closing price data is used for training NNs to predict the future price of the S&P 500. In this case, no more than approximately 250 data points can be obtained per year for training NNs since the S&P 500 is the only stock index data used for training. In this paper, we propose a simple but effective method for training NNs to predict the future price of the S&P 500 which is one of the most commonly followed stock index. In our training process, we do not use S&P 500 data for training the NNs used in our study, even though the NNs are used for predicting the future price of the S&P 500 in the test stage. Rather, we use the data of individual companies for training the NNs, as similarly done in other individual stock price prediction studies. NNs used in our study are fed with the past W days of data of individual companies and trained to predict a corresponding individual company’s price change of the next day. In the test stage, the trained NNs are fed with the past W days of data of ≈500 companies listed in the S&P 500, and the next day prediction of the S&P 500 is made based on the past W days of data of ≈500 companies.

Using the data of individual companies instead of the data of stock indexes for training NNs to predict the future price of the S&P 500 has two advantages. First, we can easily address the data-shortage problem and thus avoid overfitting, which is one of the major problems in machine learning, and commonly occurs when training complex models on a small amount of data. Second, by using the data of individual companies, we can directly use the price data of individual companies, which is generated from the investment activities of numerous investors. But if we use only the data of stock indexes for training NNs, such data is unavailable because the price of a stock index is usually the weighted average of the market capital of constituent companies and the price is not directly yielded by investors. Therefore, using the price data generated from investors’ activities can help NNs learn richer representations of the investment activities and perform better in the testing stage.

In our experiments, we compared NNs trained on the data of individual companies and trained on the data of S&P 500 index. Different types of NNs, such as Multilayer Perceptron (MLP) and Convolutional Neural Networks (CNNs), were trained using different learning algorithms such as supervised learning (SL) and reinforcement learning (RL) for comparison. It would be ideal to conduct comparison experiments using all the types of NNs or learning algorithms. But it is infeasible since there are countless types of NNs and numerous learning algorithms. Yet, our experiments in which the same learning algorithms, types of NNs, and input features are used empirically show that the NNs trained on the data of individual companies outperform the NNs trained on the data of the S&P 500. The NNs trained on only the data of individual companies also outperform the NNs in the study by [13].

The main contributions of our work are as follows. First, we propose a novel method for training NNs to predict the future price of the S&P 500. Our method uses only the data of individual companies as training data to obtain a sufficient amount of data. With our method, we can train NNs on a large amount of data, and as a result, effectively address the problems due to training NNs on a small amount of data. Second, in our experiments, we empirically show that when building NNs for stock index prediction, training the NNs on the data of individual companies is more effective than training on the data of stock index. Finally, we consider transaction costs in our experiments and introduce a simple method for controlling the number of transactions.

## Background

In this section, we briefly discuss two basic NNs used in our experiments, which are the core architectures of current state-of-the-art applications in broad areas such as NLP, image classification, text generation, speech recognition, question answering, and financial time series analysis [14]. We also discuss two basic learning methods in machine learning, which are used to train NNs in our experiments.

MLP has the most simple and basic NN structure and it is also commonly called a fully connected NN. MLP typically consists of an input layer, several hidden layers, and an output layer. Each hidden layer takes an output vector of the previous layer as input, and outputs a vector which is inputted to the next layer. The input layer takes an input feature vector as input, and the output layer usually outputs one-hot vector whose size is equivalent to the number of classes in classification problems. Each hidden layer consists of linear matrix multiplication and a nonlinear activation function. Vanilla MLP may be sufficient to solve some simple problems, but in most recent works, MLP is used as part of a more complicated structure.

CNN is widely used in image classification problems. Unlike MLP, CNN is designed to take multiple vectors or matrices as input, which makes it suitable for 2D image processing. In practice, it usually takes 2D images with three color channels as input. But it could also take 3D or 1D images as input. The core layers of CNN are as follows: convolutional layers followed by a nonlinear activation function and pooling layers. Such stacked layers enable CNN to extract high-level features from raw input images. However, recent works [15, 16] have shown that stacking more convolutional layers helps to increase the performance of CNN models. Thus recent CNN models have much more complex and sophisticated structures.

SL involves giving explicit answers to a model. If we want to use SL for training vanilla CNNs to classify handwritten digits (from 0 to 9) in a raw pixel image, the raw pixel image and its correct label are given to the CNNs during training. The CNNs are trained to learn the relationship between the input feature (raw pixel image) and label. In some cases, instead of categorical labels, numerical values are more suitable as an answer. In this case, regression can be applied to train models, but the core idea is the same as when using a label for an answer. An input feature and the correct answer are provided to a model which is trained to learn the relationship between the input feature and answer.

RL is another type of machine learning method widely used in sequential decision making research areas such as game playing, robotics, or stock prediction [17]. In RL, an agent is trained to choose the best action that would yield maximum cumulative rewards given the current state. When training an agent using RL, a new episode (training sample) is generated while the agent continues to perform actions and receive rewards.

Among different types of RL, we used Q-learning [18] in our experiments. In Q-learning, an agent uses an action value which is an expected cumulative reward of the corresponding action. During training, an agent is expected to learn an optimal action value and when the training is finished, the agent can simply choose the action with the maximum value.

## Materials and methods

In this section, we introduce a novel method for training NNs to predict the future prices of stock indexes. Unlike previous works, we do not use stock index data; we use only the historical daily closing price and volume data of individual companies for training the target NNs. There are two stages in our framework: Training Stage and Test Stage. In the training stage, daily closing price data and volume data of individual companies are fed into the target NNs. In the test stage, our trained target NNs take daily closing price data and volume data of S&P 500 companies as input, and output ≈500 predictions, each of which corresponds to the individual prediction of each constituent company in the S&P 500. The ≈500 predictions are aggregated into a single scalar value and the final prediction of the S&P 500 is made based on the scalar value.

In the training stage, only the data of individual companies is used for training the target NNs to predict the future prices of the S&P 500. Therefore, in the Experimental section, we compare the performance of NNs trained on the data of individual companies with that of NNs trained on S&P 500 data. Two different types of NNs (MLP, CNN) and learning algorithms (SL, RL) with two types of input features (closing price only, closing price and volume) are used in our experiments. Thus, a total of eight different target NNs (2×2×2) with different combinations of NNs, learning algorithms and input features are used in our experiments. All target NNs are trained (1) on the data of individual companies (our method) and (2) the data of the S&P 500 (baseline method). By comparing the price prediction performance of our proposed method and that of the existing method, we empirically show that training NNs on the data of individual companies is more effective than training on S&P 500 data.

### Data

We downloaded the daily closing price and volume data of individual companies and of the S&P 500 collected over roughly a 22-year period (1996-2018) from Yahoo Finance (https://finance.yahoo.com/). We downloaded not only the available data of the S&P 500 constituent companies but also the data of the Russel 3000 Index constituent companies to obtain more data of individual companies for training the target NNs. Note that we downloaded only the data of the S&P 500 companies that made their data available in January 2018. Besides historical closing price and volume data, the historical weights of companies in the S&P 500 were also downloaded from http://siblisresearch.com/data/weights-sp-500-companies. Since constituents of the S&P 500 and weights of companies change over time, the exact list of the constituent companies and their weights were downloaded and used in our experiments.

For our experiments, the entire data set was divided into the training set, the validation set, and the test set. The training set was used for training the target NNs. The validation set was used for hyper-parameter tuning, and the test set was used for testing and comparing the performance of our method with that of the baseline. Table 1 shows the training, validation, and test periods for our method and the baseline. For our method, the data of individual companies collected over four years was used as training data. However, for the baseline, the data of the S&P 500 collected over eight years was used as training data. The entire test period was 12 years (2006-2018) and the training and validation periods were updated every four years. Thus, the target NNs were reinitialized and retrained every four years.

**Table 1:** Training, validation, and test periods.

Method	Training	alidation	Test
Ours	2000—2004	2004—2006	2006—2010
	2004—2008	2008—2010	2010—2014
	2008—2012	2012—2014	2014—2018
Baseline	1996—2004	2004—2006	2006—2010
	2000—2008	2008—2010	2010—2014
	2004—2012	2012—2014	2014—2018

Table 2 shows the data (individual companies and the S&P 500) used for generating the input data (input x and answer y) fed into the target NNs. For our method, when generating the training and validation sets, only the data of individual companies are used. When generating the test set for our method, the data of individual companies are used for generating input x, and the data of the S&P 500 is used for generating answer y. For the baseline, all the input data for the training, validation, and test sets are generated from the data of the S&P 500. The process used to generate the input data is described in the following section.

**Table 2:** The data used for generating input x and answer y. “IND” and “S&P500” denote the data of individual companies and the data of the S&P 500, respectively.

Method		Training	Validation	Test
Ours	Input x	IND	IND	IND
	Answer y	IND	IND	S&P500
Baseline	Input x	S&P500	S&P500	S&P500
	Answer y	S&P500	S&P500	S&P500

### Input and answer

**Fig 1:**
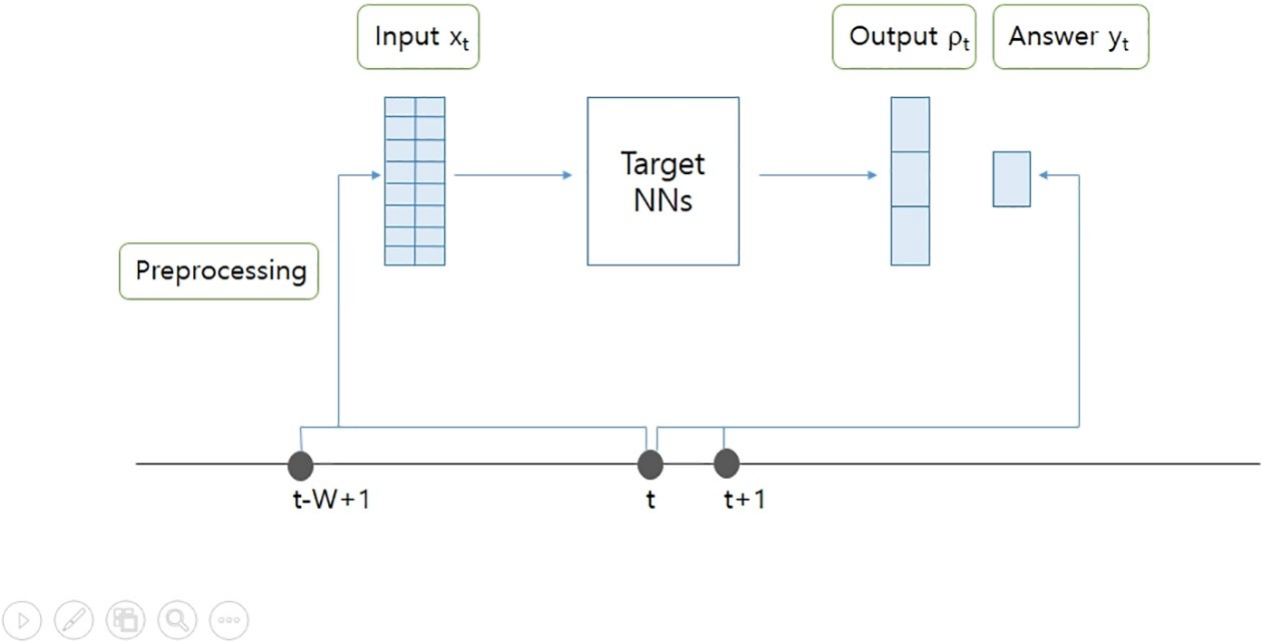
Overview of how the target NNs read input and output ρ_t_.

For our experiments, two different types of NNs and learning algorithms were utilized to validate our method. The shape of inputs and answers fed into the target NNs vary based on the network and learning algorithm used. Fig 1 shows how the input and answer are fed into the target NNs in the training process. The target NNs read input x_t_ and output ρ_t_ at time t. The answer y_t_, generated from the data of time t and t+1, evaluates the output of the target NNs ρ_t_ at time t. Table 3 summarizes how the shape of input x_t_ and the shape of answer y_t_ differ depending on the target NN and learning algorithm used.

**Table 3:** Eight different target NNs with different possible combinations of types of NNs (NNs), learning algorithms (Algs) and input features (Features). The notation of each target NN is listed in the first column. The shape of input x_t_ and the shape of answer y_t_ are listed in the last two columns, respectively. The shape (W) denotes a vector with a length of W, and the shape (W,W) denotes a W by W matrix.

Notations	NNs	Algs	Features	Shape x_t_	Shape y_t_
*MS*_*P*_	MLP	SL	Price	(W)	(3)
*MS*_*PV*_	MLP	SL	Price, Volume	(2W)	(3)
*MR*_*P*_	MLP	RL	Price	(W)	(1)
*MR*_*PV*_	MLP	RL	Price, Volume	(2W)	(1)
*CS*_*P*_	CNNs	SL	Price	(W, W)	(3)
*CS*_*PV*_	CNNs	SL	Price, Volume	(W, W)	(3)
*CR*_*P*_	CNNs	RL	Price	(W, W)	(1)
*CR*_*PV*_	CNNs	RL	Price, Volume	(W, W)	(1)

**Fig 2:**
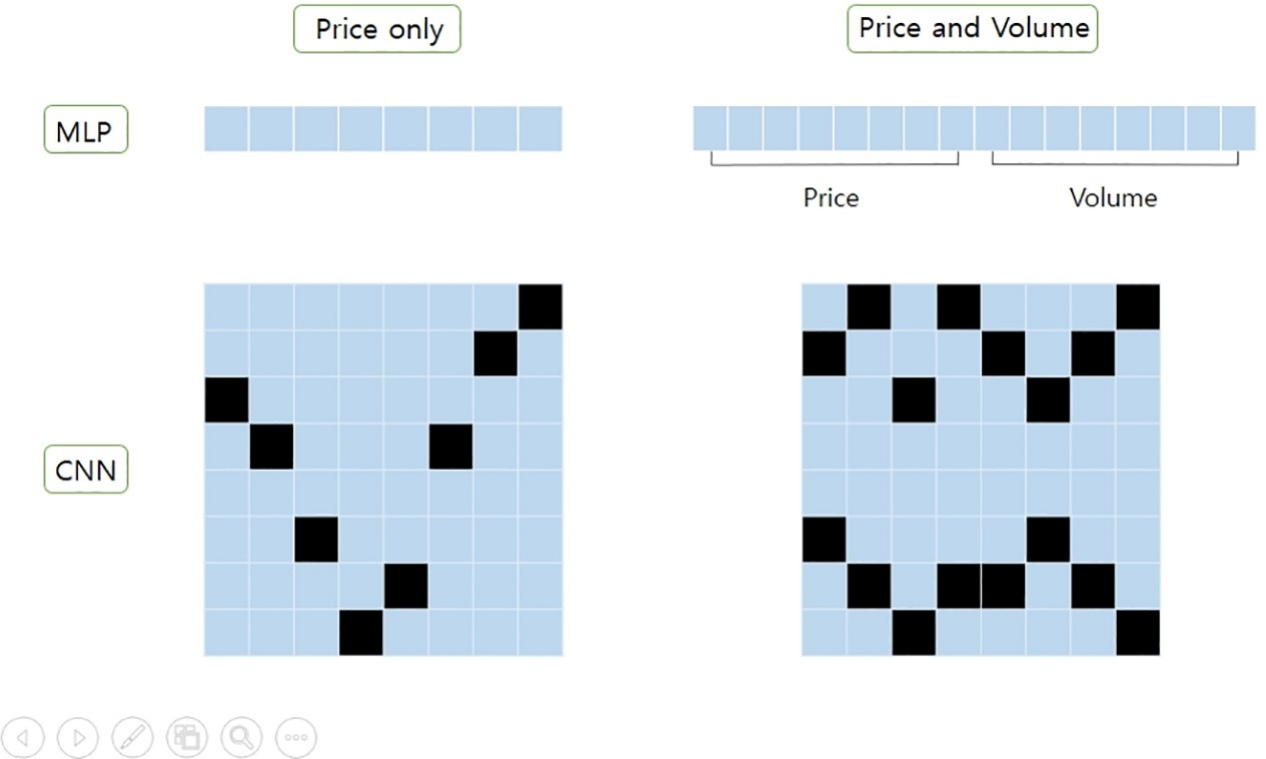
The shapes of input x_t_ for MLP and CNN, respectively.

Fig 2 illustrates the shape of input x_t_ for each of the target NNs. For MLP, input x_t_ is a vector with values min-max normalized over the last W days. The length of the vector is W when only the closing price data is used as the input feature, and the length of the vector is 2×W when both closing price and volume data are used. For CNN, a W by W matrix, which can be used as a stock chart image, is used as input x_t_. We used the same method proposed in [19] to create an image-style input matrix for CNN. In the matrix, black cells indicate the value 1 and the non-black cells indicate zero. The value 1 in the matrix indicates either relative closing price data or both relative closing price and volume data. When both closing price and volume data are included in the matrix, prices are indicted in the upper half (rows 1 to 3) and volume in the lower half (rows 5 to 8). The two rows in the middle are always empty (filled with zeros) to help CNN to distinguish closing price data from volume data. The size of the matrix is always W by W with channel size 1. This matrix can be processed as a stock chart image covering the past W days, with price indicated in the upper half and volume in the lower half, when using both closing price and volume data as input. Since CNN is widely used for image classification problems, we decided to use image-style input rather than raw numeric values for CNN.

When the vector and matrix are generated, the values of the closing price and volume data are also min-max normalized over the last W days. The min-max normalization is described in Eq 1. Only closing price is included in the equation but the equation is also applied to volume data. Note that the normalization process is done for each company in the data set over the last W days, and not over the entire training period.

$$\begin{array}{c} \tilde{P_{t}^{i}}=\left(P_{t}^{i}-P_{s}^{i}\right)/\left(P_{b}^{i}-P_{s}^{i}\right) \end{array}$$ (1)

where P^i^_b_ ≠ P^i^_s_, and $\tilde{P_{t}^{i}}$ indicates the min-max normalized value of the closing price of company i at time t. Subscripts b and s indicate the indexes of the biggest and smallest values, respectively, from time t-W+1 to time t (W days). Therefore, b, s ∈{*t* − *W* + 1, …, *t*}. If the values of P^i^_b_ and P^i^_s_ are the same, 0.5 is assigned to $\tilde{P_{t}^{i}}$.

In our experiments, the output of the target NNs is always a vector ρ_t_ with a length of 3 where elements correspond to Long, Neutral and Short positions, respectively. In other words, the target NNs can take either a Long, Neutral, or Short position. Therefore, in SL, the three positions are considered as three classes, and in RL, the three positions are considered as actions.

As input x_t_, the method used for generating answer y_t_ can vary depending on the learning algorithm used. In SL, labels are assigned to the target NNs in the training stage, and y_t_ is a one-hot vector with a length of 3 where each element corresponds to one of the three classes: Long, Neutral, or Short. In addition, the labels were equally divided between the training set and validation set by the following process. First, the training data is sorted based on daily returns in descending order. Then, for the top 33.33%, [1, 0, 0] is assigned to vector y_t_, for the bottom 33.33%, [0, 0, 1] is assigned to vector y_t_, and for the median 33.33%, [0, 1, 0] is assigned to vector y_t_.

In RL, instead of the labels, the rewards are given to the target NNs. The reward is calculated based on the output of the target NNs *ρ*, and the daily return at time t+1. Thus in RL, y_t_ is a scalar value which is the daily return at time t+1. Eq 2 defines the daily return at time t+1.

$$\begin{array}{c} D_{t+1}^{i}=100\times \left(P_{t+1}^{i}-P_{t}^{i}\right)/P_{t}^{i} \end{array}$$ (2)

where P^i^_t_ indicates the closing price of company i at time t. As in SL, the training set and validation set are also neutralized. In other words, the average value of the daily returns from each set is subtracted from each daily return. By doing this, the sum of the daily returns of the training set and validation set is zero.

To address the data imbalance problem, the training set and validation set are neutralized in RL, and the labels are distributed equally in SL. Since stock market data has more positive values than negative values, which can be attributed to the fact that the overall economy has grown over the last several decades (or longer), naively using imbalanced data as the training set may cause the model to output only the single label (Long) or perform well only when the overall market tends to be bullish.

### Training stage

In the training stage, the target NNs are trained on the historical closing price and volume data. As shown in Table 3, eight different target NNs with possible combinations of NNs, learning algorithms and input features are trained respectively. For our method, these eight target NNs are trained on the data of individual companies. For the baseline, eight target NNs are trained on S&P 500 data.

The overall process of feeding and training the target NNs in the training stage is shown in Fig 1. The target NNs are trained to predict the return at time t+1 (price change from time t to t+1 in percentage) based on the past W days of data which can be observed at time t. The output of the target NNs is a vector ρ_t_ with a length of 3, where three elements correspond to Long, Neutral, and Short positions, respectively.

When SL is used for training, the final layer of the target NNs is the softmax layer. Therefore, each element of ρ_t_ represents the probability of the input x_t_ being classified as its corresponding label (Long, Neutral, or Short). The cross-entropy loss defined in Eq 3 is used for training the target NNs. The subscript for timestep and the superscript for company are omitted for simplicity.

$$\begin{array}{c} {\text{Loss}}_{S}=\sum -y\circ \;\text{log}\;\rho \end{array}$$ (3)

where ∘ represents element-wise multiplication and y is a one-hot vector with three elements representing Long, Neutral, and Short positions, respectively. The summation is calculated over each of the randomly sampled mini-batch sizes of β. The training algorithm is described in Algorithm 1.

As mentioned in the Introduction section, for RL, we used Q-learning as our training algorithm. We adopted the methods of using experience replay and periodically updating target parameter proposed in [20] to stabilize our training process. But when performing the gradient step, we used the modified version of experience replay proposed in [19] to include more companies in one mini-batch.

In Q-learning, each element of ρ_t_ represents its corresponding action value which is the expected cumulative reward of the action. Therefore, when the training is finished, the optimal behavior of the target NNs is simply choosing the action with the maximum action value. To train the target NNs using RL, we first need to define the reward function.

$$\begin{array}{c} r_{t}^{i}=a_{t}^{i}\times D_{t+1}^{i}-P\times \left|a_{t}^{i}-a_{t-1}^{i}\right| \end{array}$$ (4)

where a^i^_t_ is a scalar value that represents the chosen action of company i at time t. The values 1, 0, and -1 are assigned to a^i^_t_ for Long, Neutral, and Short actions, respectively. P is the transaction penalty used during the training to prevent the target NNs from changing their position too frequently.

For our training algorithm, we use the same loss function proposed in [20]. The loss function utilizes the Bellman equation which defines the relationship between the action value at the current time step and that at the next time step, and iteratively updates the action value until it converges to the optimal action value. The loss function is defined below. The subscript for time step and the superscript for company are omitted for simplicity.

$$\begin{array}{c} {\text{Loss}}_{R}=\sum {\left[r+\gamma \underset{a^{\prime }}{\text{max}}Q\left(s^{\prime },a^{\prime };\theta^{*}\right)-Q\left(s,a;\theta \right)\right]}^{2} \end{array}$$ (5)

where *γ* denotes the discount factor, and s, a, r, s’ and a’ represent the current input x_t_, the chosen action a_t_ given the current input x_t_, the immediate reward r_t_, the subsequent input x_t+1_, and the next action a_t+1_ given input x_t+1_, respectively. Q(s,a;θ) which is parameterized by *θ* denotes the action value of the chosen action given the current input s. For example, if the chosen action a_t_ is Long given the current input x_t_, then Q(s,a;θ) is exactly equal to ρ_t_[0]. When choosing an action given the current input x_t_, the *ε*-greedy policy is used. The *ε*-greedy policy chooses the action with the maximum action value with a probability of 1-*ε* or chooses a random action with a probability of *ε*.

To apply experience replay, at every iteration, we store our randomly sampled experience e_b_ = {s,a,r,s’} in the memory buffer with the size of M. Then the mini-batch size of β is randomly sampled from the memory buffer at every B iteration to perform the gradient step for minimizing Loss_R_, with respect to the parameter θ. Thus, the summation in Eq 5 is calculated over each of the mini-batch sizes of β. The two parameter sets θ and θ* are maintained throughout our training to avoid the moving target problem. The parameter θ* is only updated every B×C iteration by simply copying the parameter θ to θ*. The training algorithm is described in Algorithm 2.

### Training details

In this subsection, we will briefly discuss the training details, such as how we chose the hyper-parameters and the best performing model parameter θ. First of all, we chose the optimal hyper-parameter values by repeating the training-validation process on the training and validation sets, each from 2000-2004 and 2004-2006, respectively. We mostly conducted grid search to select the values rather than random searching. The values of hyper-parameters are listed in Table 4. The network structure selected in this stage is highlighted in Table 8. While constructing the network structure, batch normalization layers [21] are added after each layer for both MLP and CNN. The Adam optimizer [22] is used to perform a gradient step on Loss_S_ and Loss_R_. Once the hyper-parameters and the network structures are selected in this stage, those selected hyper-parameters and network structure are used for entire test period both for our method and the baseline.

**Table 4:** List of hyper-parameters and their values used while training.

Algo	Notations	Description	Values
SL	β	The batch size	64
	*maxiter*	The maximum number of iterations	200,000
	*learning rate*	The learning rate	0.001
RL	M	The size of the memory buffer	1,000
	B	The update interval of parameters θ	10
	C	The update interval of parameters θ*	1,000
	P	The transaction penalty	0.1
	β	The batch size	32
	*γ*	The discount factor	0.99
	*maxiter*	The maximum number of iterations	3,000,000
	*learning rate*	The learning rate	0.00001

Next, we chose the optimal model parameter θ as follows. In the training stage, we store parameter θ every 0.1 × *maxiter* iterations and evaluate the parameter on the validation set rather than simply using the parameter θ after *maxiter* iterations. When training is finished, the parameter that obtained the best performance on validation set is selected and used for the test set. The process of obtaining optimal parameter θ is carried out for each validation period. For example, the validation set from period 2012-2014 is used to choose the optimal parameter θ for the test set from period 2014-2018. The same process is used for the baseline for fair comparison.

### Test stage

**Fig 3:**
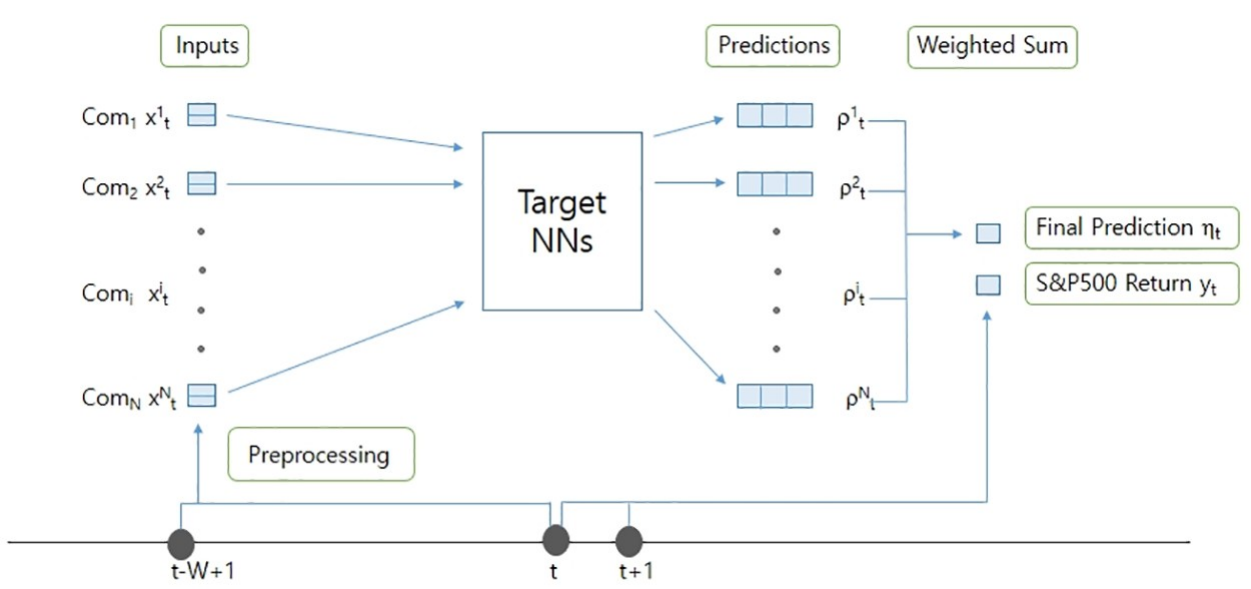
Overview of how η_t_ is aggregated from the outputs of individual companies ρ^i^_t_.

In the test stage of our method, the eight target NNs with two NNs, different learning algorithm and input feature combinations trained on the data of individual companies were tested. The target NNs trained on the data of the S&P 500 were also tested as a baseline. In this subsection, the exact method of aggregating the output of the target NNs trained on the data of individual companies will be discussed. Fig 3 shows how the target NNs aggregate the predictions of individual companies and make the final prediction *η*_t_ of the future price of the S&P 500 at time t.

The target NNs take input generated from every constituent company of the S&P 500 over the last W days at time t, and predict the future price of the S&P 500 at the subsequent time step. In other words, the target NNs decide which optimal position [Long, Neutral, Short] should be taken at time t based on all the constituent companies. For the remainder of this paper, we use N as the number of constituent companies of the S&P 500 used in our experiments. Even though the S&P 500 has 505 constituent companies, we use N to denote the number of constituent companies because we were unable to obtain the data of some constituents. Therefore, the value N varies depending on the experimental period.

The final prediction η_t_ is calculated as follows. First, at time t, the target NNs independently take input x^i^_t_ N times, and independently output vectors ρ^i^_t_ N times. Each vector ρ^i^_t_ represents the prediction made by a target NN for company i at time t. In SL, each element in vector ρ^i^_t_ represents the probability of the input x^i^_t_ being classified as its corresponding label. In RL, each element in vector ρ^i^_t_ represents the action value of its corresponding action. Therefore, in SL and RL, the subtracted value (ρ^i^_t_[0]—ρ^i^_t_[2]) of each company is calculated for representing the probability of price of company i rising at subsequent time step t+1. The final prediction η_t_ is a weighted sum of the subtracted values each of which weighted by the market capitalization of the corresponding companies at time t. Whether to take a Long, Neutral, or Short position in the S&P 500 at time t is decided based on the value of η_t_. For example, we can take a Long position in the S&P 500 if η_t_ is bigger than 0; if η_t_ is smaller than 0, we can take a Short position at time t. The exact equation for calculating η_t_ is as follows.

$$\begin{array}{c} \eta_{t}=\sum_{i=0}^{N}Cap_{t}^{i}\times \left(\rho_{t}^{i}\left[0\right]-\rho_{t}^{i}\left[2\right]\right) \end{array}$$ (6)

where Cap^i^_t_ represents the market capital ratio of company i at time t and satisfies ($\sum_{i=0}^{N}$ Cap^i^_t_ = 1.0).

In most cases, when using the final prediction value η_t_ to decide which position should be taken in the S&P 500 at time t, the average value of η_t_ is not zero. In other words, the strategy of taking a Long position in the S&P 500 when η_t_ is bigger than 0 or a Short position when it is smaller than 0 may lead to taking either a Long or Short position too frequently. To balance the two positions, we calculate the mean μ_η_ and standard deviation σ_η_ of η_t_ over each validation set. We use the mean μ_η_ and standard deviation σ_η_ to decide which position to take in the S&P 500. For example, we can take a Long position in the S&P 500 when η_t_ is bigger than μ_η_ + σ_η_ or a Short position when η_t_ is smaller than μ_η_—σ_η_. The exact method for deciding which position to take in the S&P 500 using μ_η_ and σ_η_ is described in Algorithm 3 and will be discussed in the next section.

**Algorithm 1**: Supervised Learning

1: Initialize parameter θ

2: **for all** *b = 0, maxiter*

3:  Random sample mini-batch of size β from training data

4:  Calculate Loss_S_ in mini-batch

5:  Perform gradient step to minimize Loss_S_ with respect to the parameter θ

6:  **if** b% (0.1 × *maxiter*) == 0 **then**

7:   Store current parameter θ and obtain performance on validation set.

8:  **end if**

9: **end for**

**Algorithm 2**: Reinforcement Learning

1: Initialize the memory buffer to capacity M

2: Initialize parameter θ

3: Initialize parameter θ* ← θ

4: **for all** *b = 0,maxiter* **do**

5:  Random sample current state s from the training set

6:  With ε-greedy policy, choose action a, given the current state s

7:  Observe immediate reward r and next state s’

8:  Store experience e_b_ = {s,a,r,s’} in the memory buffer

9:  **if** b% B == 0 **then**

10:   Random sample mini-batch of size β from the memory buffer

11:   Calculate Loss_R_ in mini-batch

12:   Perform gradient step to minimize Loss_R_ w.r.t. the parameter θ

13:  **end if**

14:  **if** b% (B×C) == 0 **then**

15:   Set θ* ← θ

16:  **end if**

17:  **if** b% (0.1 × *maxiter*) == 0 **then**

18:   Store current parameter θ and obtain performance on validation set.

19:  **end if**

20: **end for**

## Experiments

### Comparison with baseline

In this subsection, the experimental results of the eight different target NNs with various combinations of types of NNs, learning algorithms and input features are provided. The target NNs were (1) trained on the data of individual companies and the S&P 500 and tested on the data of the S&P 500. The notations of the eight target NNs are provided in Table 3. In this paper, we are proposing a new method, rather than a unique neural network structure or a learning algorithm, for training neural networks for stock index prediction. The comparison of our method and existing method demonstrates that training target NNs on a large amount of data of individual companies is more effective in improving performance than changing the network structure or learning algorithm.

**Fig 4:**
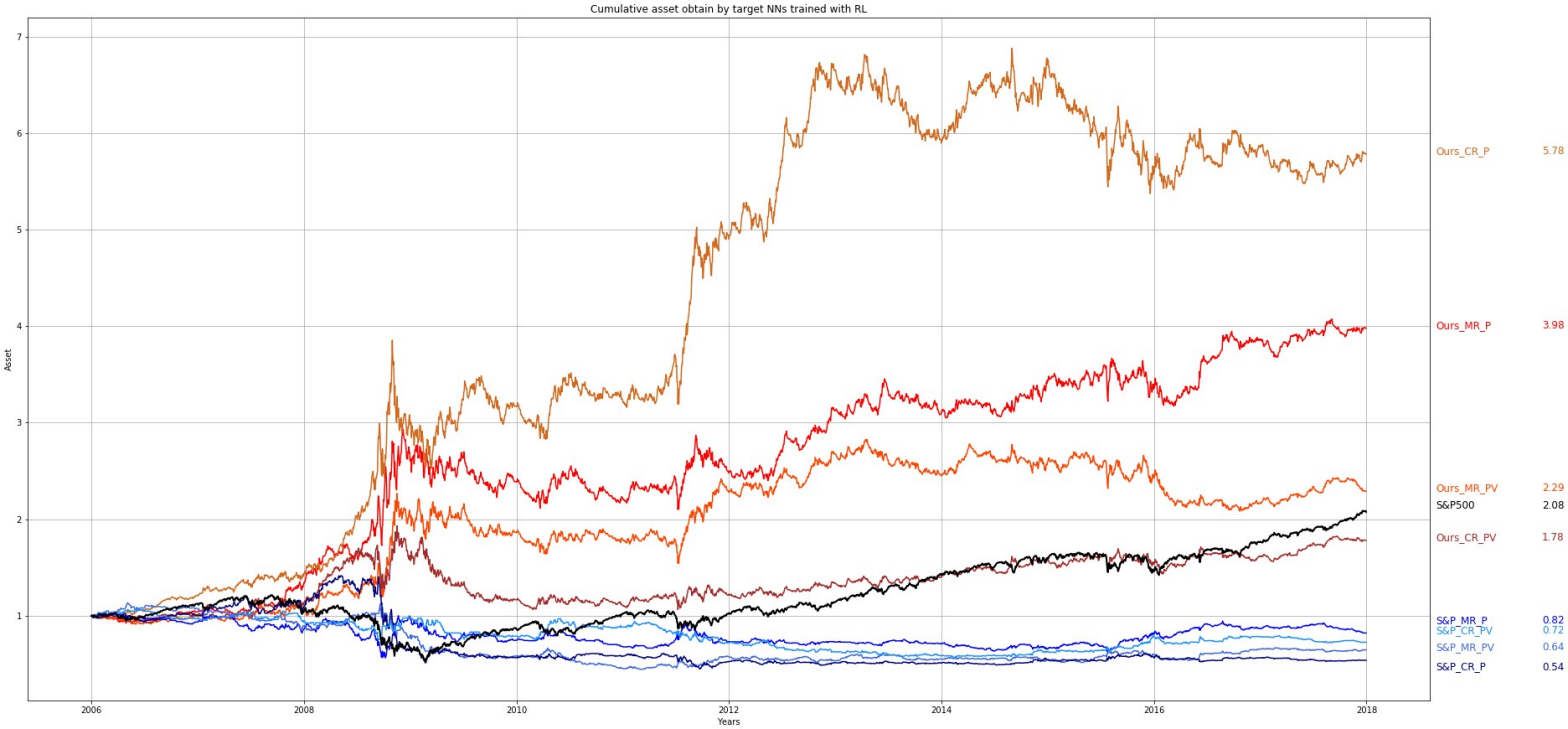
Cumulative assets obtained by target NNs trained using RL. Cumulative assets obtained over the entire test period. The notations and final assets are listed at the right side of the figure.

**Fig 5:**
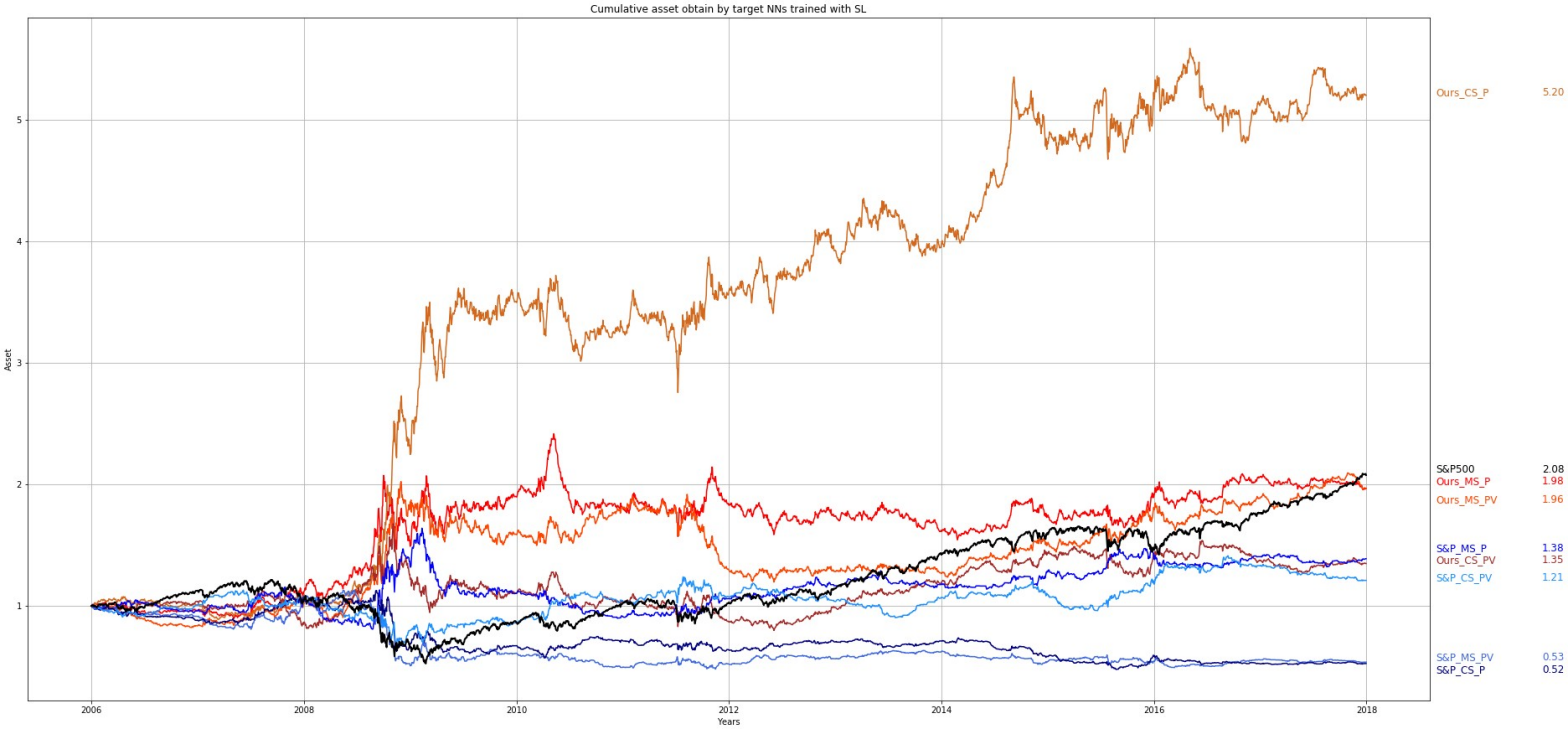
Cumulative assets obtained by target NNs trained using SL. Cumulative assets obtained over the entire test period. The notations and final assets are listed at the right side of the figure.

Figs 4 and 5 show the cumulative assets obtained by each of the eight different NNs throughout the entire test period (12 years). For visualization purposes, the results are divided and shown in the two figures. In Fig 4, the experimental results of four target NNs trained using RL are provided. In Fig 4, for our method, the target NNs trained on the data of individual companies are labeled with the prefix “Ours” and for the baseline method, the target NNs trained on the data of the S&P 500 are labeled with the prefix “S&P.” In Fig 5, the experimental results of the target NNs trained using SL and our method and the baseline method are provided. The cumulative asset obtained by the buy-and-hold strategy of the S&P 500 is labeled as “S&P 500” and shown in Figs 4 and 5. For each NN, the asset is assumed to be 1.0 at the initial point. Every four years, the parameters of the target NNs are replaced with re-trained parameters and used for the subsequent four years. The transaction cost is not considered in this experiment.

As shown in Figs 4 and 5, most of the target NNs trained using RL and SL and trained on the data of individual companies outperforms the target NNs trained on the data of the S&P 500. It is difficult to say whether MLP or CNN is better for predicting the future price of the S&P 500. Also, adding the volume data for input features does not help improve the performance of the target NNs. But the target NNs trained using RL performs better than the target NNs trained using SL when the data of individual companies are used for training.

Figs 4 and 5 show that when the same learning algorithms and input features are used, the target NNs trained on the data of individual companies outperforms the target NNs trained on the data of the S&P 500 in predicting the future price of the S&P 500. The results also show that training the target NNs on a sufficient amount of data of individual companies is more effective than changing the network structure or learning algorithm for improving performance.

### Comparison with previous work

In this subsection, we compare our method with a state-of-the-art method [13] that adopts deep Q-learning and transfer learning [23] to predict the future prices of stock indexes and determine the number of shares to trade. In their experiments, the authors either chose 6 or 10 constituent companies in each stock index, and used the price data of these constituent companies for pretraining. After the pretraining, the price data of each stock index was used for fine-tuning. The authors conducted four independent experiments on the following four stock indexes: S&P 500, KOSPI, HSI, and EuroStoxx50. But we considered only the experimental results on the S&P 500 since our experiments are conducted on only the S&P 500. Also, since determining the number of shares to trade is not the main focus of our work, we did not compare their experiments with ours.

Among our eight target NNs reported in the previous subsection, we chose the following four target NNs for the experiment: CR_p_, MR_p_, CS_p_, and MS_p_. Since the model proposed in previous work [13] used only daily price data as input, we chose four NNs that use only price data as input. Also, we recalculated the profit from the same test period (Jun. 5, 2006-Dec. 29, 2017) used in the previous work [13]. We also used the same evaluation metric used in the previous work, which is defined in Eq 7.

$$\begin{array}{c} {\text{Profit}}_{t}=a_{t}\times \left(P_{t+1}-P_{t}\right)/P_{t} \end{array}$$ (7)

where P_t_ is the closing price of the S&P 500 at time t and a_t_ is a position taken at time t. The superscript i for a company is omitted for simplicity. The values 1, 0, and -1 are assigned to a_t_ for Long, Neutral, and Short actions, respectively. Thus, for example, a profit of 1.0 at the end of the test period could be interpreted as a 100% asset gain over the entire test period assuming that the profit earned from the trade is not reinvested.

Table 5 compares the profits gained by our method and those gained by the method proposed in [13]. For better understanding, we will briefly explain the notations used in Table 5. In the previous work of [13], the constituent companies were chosen based on the past price sequence similarity between the constituent companies and the stock indexes. The authors used correlation and NNs to measure the past price sequence similarity. In their work, the notations CR and NE denote correlation and NN, respectively. The subscripts H, HL, and L denote high, high and low, and low, respectively. Thus, for example, “CR_H_” denotes a model pretrained on the data of the constituent companies that have a high correlation with the stock index. As shown in Table 5, on average, our method outperforms the method in [13]. The two target NNs that use CNN (CR_p_ and CS_p_) yielded more profit than the best performing NE_L_ from the previous work.

**Table 5:** Comparison of the total profit gained by our method and that obtained by the method proposed in [13]. The profit is summed over the entire test period (Jun. 5, 2006-Dec. 29, 2017). “Average” denotes the average profit.

	Notations	Profit
Ours	*CR*_*P*_	**1.782**
	*MR*_*P*_	1.527
	*CS*_*P*_	**1.956**
	*MS*_*P*_	0.869
	**Average**	**1.533**
Jeong & Kim	*CR*_*H*_	0.925
	*CR*_*HL*_	1.039
	*CR*_*L*_	1.431
	*NE*_*H*_	1.292
	*NE*_*HL*_	1.076
	*NE*_*L*_	**1.595**
	**Average**	**1.226**

### Considering transaction cost

In this subsection, we discuss how to use our method in real practice. We conducted an experiment considering transaction costs and adopting other financial indicators besides cumulative assets. In the subsection Comparison with Baseline, we compare the cumulative returns of the target NNs trained on the data of individual companies with those of NNs trained on the S&P 500 data to show that our method is more effective in training NNs for stock index prediction. However, in real practice, it is important to also consider transaction costs and other indicators such as annual return, Maximum Drawdown [24], the number of transactions, and the ratio of Long to Short positions.

Table 6 shows additional information obtained by the eight target NNs trained on the data of individual companies (our method) and the data of the S&P 500 (baseline method). The results of the same target NNs used in the previous subsection are provided in Table 6. As shown in Table 6, the target NNs trained on the data of individual companies earned annual returns of about 5%-15%, which are much higher than the annual returns of the target NNs trained on the data of the S&P 500. Typically, the ratio of Long to Short positions is slightly less than 0.5. Training the target NNs on a neutralized training set and using the mean μ_η_ of η_t_, calculated over each of the validation sets, helped balance the ratio of Long to Short positions. Also, the profits per transaction are mostly around 0.05%-0.2%, except for CR_P_. As listed in column TR in Table 6, the number of transactions of the target NNs trained using RL is less than that of the target NNs trained using SL, due to the transaction penalty which is applied in the training stage of RL and described in Eq 4.

**Table 6:** Comparison of the annual returns and returns per transaction of our method and those of the baseline. The columns Return, TR, Long, and perTR list the annual returns in percentage, the number of transactions per year, the Long to Short position ratio, and the returns per transaction, respectively. The results are averaged over the entire test period.

	Notations	cumAsset	Return (%)	TR	Long	perTR (%)
Ours	CR_P_	5.78	17.04	37.99	0.46	0.45
	CR_PV_	1.78	6.89	42.4	0.42	0.16
	MR_P_	3.98	13.82	58.96	0.44	0.23
	MR_PV_	2.29	9.05	51.8	0.42	0.16
	CS_P_	5.20	16.12	91.05	0.47	0.18
	CS_PV_	1.35	4.49	89.85	0.47	0.05
	MS_P_	1.98	7.74	111.84	0.44	0.07
	MS_PV_	1.96	7.75	105.06	0.47	0.07
Baseline	CR_P_	0.54	-3.67	83.39	0.48	-0.04
	CR_PV_	0.72	-1.67	77.39	0.32	-0.02
	MR_P_	0.82	-0.16	78.46	0.43	0.0
	MR_PV_	0.64	-2.27	91.19	0.54	-0.03
	CS_P_	0.52	-4.21	97.7	0.48	-0.04
	CS_PV_	1.21	2.97	94.38	0.57	0.03
	MS_P_	1.38	4.24	101.76	0.49	0.04
	MS_PV_	0.53	-3.86	95.33	0.47	-0.04

**Algorithm 3**: Lagged Position Change

**Function** *laggedPosChange* (*η*_*t*_, *α*_*t*-1_, *μ*_*η*_, *σ*_*η*_, *τ*_*w*_, *τ*_*s*_)

 UprTH_s_ ← μ_η_ + τ_s_ × σ_η_

 UprTH_w_ ← μ_η_ + τ_w_ × σ_η_

 LwrTH_s_ ← μ_η_—τ_s_ × σ_η_

 LwrTH_w_ ← μ_η_—τ_w_ × σ_η_

 **if** *η*_*t*_ < *LwrTH*_*s*_ *or UprTH*_*s*_ < *η*_*t*_ **then**

  **if** *η*_*t*_ < *LwrTH*_s_ **then**

   return -1

 **else**

   return 1

 **else**

  **if** *LwrTH*_*w*_ ≤ *η*_*t*_ *and η*_t_ ≤ *UprTH*_*w*_ **then**

   return 0

  **else**

   **if** *η*_t_ < *LwrTH*_*w*_ **then**

    **if** *α*_*t-1*_ == *1* **then**

     return 0

    **else**

     return α_t-1_

  **else**

    **if** *α*_*t-1*_ == *-1* **then**

     return 0

    **else**

     return *α*_t-1_

However, the profits per transaction listed in Table 6 are still too small after considering the transaction cost. Therefore, we introduce Lagged Position Change which is a simple algorithm that reduces the number of transactions and increases the profit per transaction using the mean μ_η_ and standard deviation σ_η_, both of which are discussed in the Methods section (E. Test Stage). The intuition behind this algorithm is as follows. A Long position is taken when the prediction value η_t_ is certainly positive, and a Short position is taken when the value is certainly negative. When the prediction value η_t_ is weak, a neutral position is taken. If the value is somewhere between weak and strong, the same position that was taken at the previous time step is taken to prevent changing the position too frequently. Algorithm 3 describes the function ***laggedPosChange*** which returns the current position α_t_ based on the current prediction value η_t_ and the previous position α_t-1_. The return value of the function ***laggedPosChange*** α_t_ could be 1, 0, or -1 which correspond to Long, Neutral, or Short positions, respectively. The two arguments τ_w_ and τ_s_ are scalar values multiplied by the standard deviation σ_η_, which satisfies τ_w_ ≤ τ_s_. The arguments are used to determine how strong the prediction value η_t_ should be, to take a Long or Short position. In our experiment, we limited the value of τ_s_ to 0.75. If a value that is too large is assigned to τ_s_, the target NNs would take only the Neutral position in most cases. The results listed in Table 6 in the previous subsection are exactly the same as the results obtained by the function ***laggedPosChange*** with the arguments τ_w_ and τ_s_ both equal to zero.

Table 7 lists the results obtained by the function ***laggedPosChange*** with the value τ_s_ between 0 and 0.75. Only the results of the target NNs trained on the data of the individual companies (Ours) are listed. The values in the first 8 rows are the averaged results of the target NNs trained using RL (CR_P_, CR_PV_, MR_P_, and MR_PV_). For example, the cumulative asset of 3.46 in the first row is the averaged cumulative asset (5.78+1.78+3.98+2.29)/4, listed in the column cumAsset in Table 6. Each row provides the averaged result obtained by changing the value τ_s_ and transaction cost. Also, the values in the last 8 rows are the averaged results of the target NNs trained using SL (CS_P_, CS_PV_, MS_p_, and MS_PV_).

**Table 7:** The averaged results obtained using the function *laggedPosChange* for the target NNs trained on the data of individual companies (Ours). The column TRCost and the column cumAsset list the transaction costs and cumulative assets, respectively. The column NNP and the column MDD list the non-neutral position ratios and Maximum Drawdowns, respectively.

Algo	τ_w_	τ_s_	TRCost	cumAsset	return (%)	TR	perTR (%)	Long	NNP	MDD
RL	0	0	0	3.46	11.7	47.79	0.25	0.44	1	-21.93
	0	0.25	0	3.37	11.72	36.65	0.33	0.42	0.69	-18.67
	0	0.50	0	3.28	11.34	28.79	0.4	0.42	0.58	-17.39
	0	0.75	0	2.56	8.98	21.74	0.42	0.41	0.47	-16.45
	0	0	0.1	2.02	6.92	47.79	0.15	0.44	1	-24.83
	0	0.25	0.1	2.2	8.05	36.65	0.23	0.42	0.69	-21.4
	0	0.50	0.1	2.33	8.46	28.79	0.3	0.42	0.58	-19.02
	0	0.75	0.1	1.98	6.81	21.74	0.32	0.41	0.47	-17.46
SL	0	0	0	2.62	9.03	99.44	0.09	0.46	1	-25.86
	0	0.25	0	2.58	8.97	75.09	0.12	0.46	0.63	-20.18
	0	0.50	0	2.6	9.02	58.79	0.15	0.47	0.51	-18.68
	0	0.75	0	2.26	7.87	44.43	0.18	0.47	0.4	-18.63
	0	0	0.1	0.85	-0.92	99.44	-0.01	0.46	1	-34.39
	0	0.25	0.1	1.09	1.47	75.09	0.02	0.46	0.63	-26.1
	0	0.50	0.1	1.31	3.14	58.79	0.05	0.47	0.51	-21.99
	0	0.75	0.1	1.34	3.43	44.43	0.08	0.47	0.4	-21.15

As Table 7 shows, when we increase the value τ_s_, the number of transactions and the non-neutral position (column NNP) ratio decrease. The NNP measures the non-neutral position ratio which is calculated by dividing the sum of the Long and Short positions by the sum of the Long, Neutral, and Short positions. Therefore, when we increase the τ_s_, the target NNs buy or sell the S&P 500 and change the current position only when the situation is more certain. The column perTR clearly shows that increasing the value τ_s_ increases the return per transaction; the cumulative asset or annual return only slightly decreases, which may be due to the decrease in the non-neutral position ratio. Increasing τ_s_ increases both the cumulative asset and the return per transaction even more when transaction cost is applied. The target NNs did not yield positive returns especially in the SL cases where the number of transactions is quite high. Also, increasing τ_s_ also helped reduce the Maximum Drawdown.

### Robustness verification

The results of our previous experiments show that training the target NNs on the data of individual companies improves performance more than changing the learning algorithm or adding additional input features. However, the performance of the NNs is known to vary depending on their network structure such as the number of layers or the number of parameters of each layer.

In this subsection, we discuss an experiment conducted with the target NNs (MLP and CNN) with different network structures. We changed the number of layers or the number of parameters of each layer of each target NNs, and trained each target NNs using either SL or RL. As similarly done in the previous experiments, we trained the target NNs with different learning algorithms and network structures on the data of individual companies and the data of the S&P 500. But for this experiment, we used only the closing price data as input because using the volume data did not help improve the performance of the target NNs. Table 8 lists the network structure details and all the target NNs with various learning algorithms and network structures used in this experiment. Also, Table 8 lists the cumulative assets obtained by the target NNs trained on the data of individual companies (our method) and the target NNs trained on the data of the S&P 500 (baseline method) over the entire test period. We tested four different network structures including the same network structure used for MLP and CNN in the previous subsections. For example, in Table 8, MS_P2_ and MR_P2_ have the same network structure. Therefore, in total, sixteen target NNs with possible combinations of types of NNs, network structures and learning algorithms were tested in this experiment.

**Table 8:** Sixteen target NNs with different learning algorithms (Algs) and network structures. The first column lists the notation of each NN. The network structures (MS_p_, MR_p_, CS_p_, and CR_p_) used in the previous experiments are highlighted. The column Layers lists the number of fully connected layers of MLP and the number of convolutional layers for CNN. The column Units/Filters lists the number of units in the hidden layers of MLP and the number of filters in the convolutional layers of CNN. The columns Ours and Baseline list the cumulative assets obtained by the target NNs over the entire test period.

Notation	NNs	Algs	Layers	Units / Filters	Ours	Baseline
**MS_p_**	**MLP**	**SL**	**4**	**64, 32, 16, 3**	**1.98**	**1.38**
MS_P1_	MLP	SL	4	128, 64, 32, 3	2.18	1.31
MS_P2_	MLP	SL	3	64, 32, 3	6.7	2.57
MS_P3_	MLP	SL	3	128, 64, 3	4.48	1.82
**MR_P_**	**MLP**	**RL**	**4**	**64, 32, 16, 3**	**3.98**	**0.82**
MR_P1_	MLP	RL	4	128, 64, 32, 3	3.45	1.27
MR_P2_	MLP	RL	3	64, 32, 3	5.0	1.62
MR_P3_	MLP	RL	3	128, 64, 3	6.04	1.16
**CS_P_**	**CNNs**	**SL**	**4**	**8, 8, 16, 16**	**5.2**	**0.52**
CS_P1_	CNNs	SL	4	16, 16, 32, 32	2.28	1.07
CS_P2_	CNNs	SL	2	16, 32	2.72	0.38
CS_P3_	CNNs	SL	2	32, 64	1.24	0.71
**CR_P_**	**CNNs**	**RL**	**4**	**8, 8, 16, 16**	**5.78**	**0.54**
CR_P1_	CNNs	RL	4	16, 16, 32, 32	2.92	1.3
CR_P2_	CNNs	RL	2	16, 32	3.84	0.98
CR_P3_	CNNs	RL	2	32, 64	4.79	1.0

**Fig 6:**
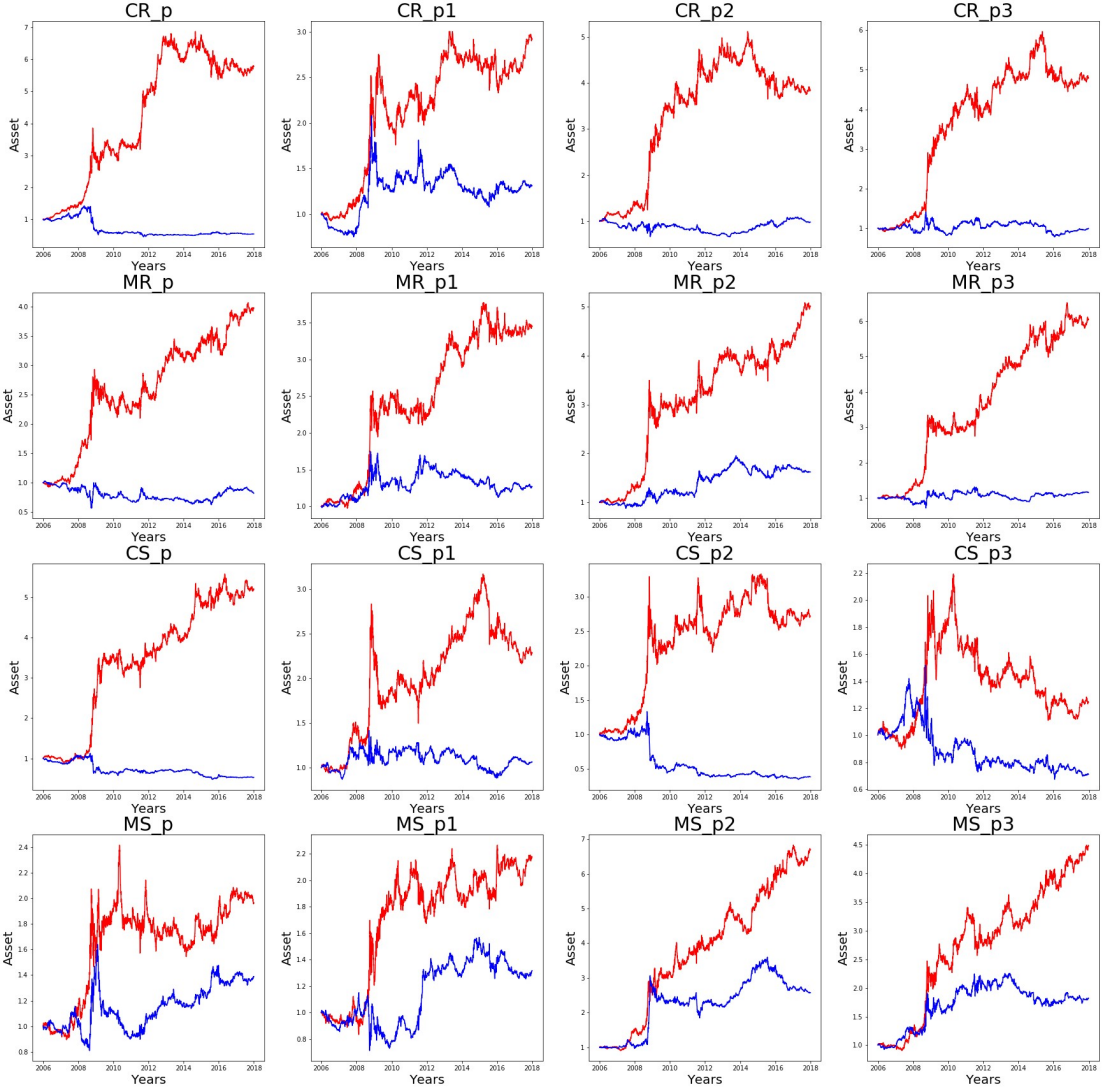
Cumulative assets over the entire test period. Comparison of the cumulative assets obtained by sixteen different target NNs. Cumulative assets obtained by the target NNs trained on the data of individual companies (our method) are highlighted in red. Cumulative assets obtained by the target NNs trained on the data of the S&P 500 (baseline method) are highlighted in blue.

Table 8 and Fig 6 compare the cumulative assets obtained by each of the 16 target NNs trained on the data of individual companies, and the same target NNs trained on the data of the S&P 500. The cumulative assets obtained by the target NNs trained on the data of individual companies (our method) are highlighted in red, and the cumulative assets obtained by the target NNs trained on the S&P 500 data (baseline method) are highlighted in blue. The performance results of the 16 target NNs are presented in 16 different graphs for comparison. Thus, for example, in Fig 6, the graph titled “MS_p2” compares the cumulative assets obtained by MS_P2_ trained on the data of individual companies with the cumulative assets obtained by MS_P2_ trained on the data of the S&P 500.

As shown in Table 8 and Fig 6, the target NNs trained on the data of individual companies mostly outperformed the target NNs trained on the data of the S&P 500. When CNNs were used as the target NNs and trained on the S&P 500 data, they did not yield profit in most cases. When MLPs were used as the target NNs and trained on the data of the S&P 500, they yielded competitive returns (MS_P2_) in some cases. However, the target NNs trained on the data of individual companies consistently outperformed the target NNs trained on the data of the S&P 500, and yielded profit regardless of the network structure, learning algorithm, or input feature used.

Theoretically, numerous network structures can be constructed. But it is infeasible to test and compare all network structures. Therefore, we chose four different network structures for the NNs and report the performance of the target NNs with different network structures. Through these experiments, we empirically verify the followings: First, when the same network structure and learning algorithm are used, the target NNs trained on the data of individual companies mostly outperform the target NNs trained on the data of the S&P 500. Second, the performance of the target NNs varies depending on their network structure. For example, in the case of CS_P3_, the cumulative asset is 1.24, but in the case of MS_P2_, the cumulative asset is 6.7. Training the target NNs on the data of individual companies obtains more consistent performance than training the target NNs on the data of the S&P 500.

## Discussion

In this work, we proposed a novel method for training various types of NNs to predict the future price of the S&P 500, one of the most commonly traded stock indexes. Unlike previous works, we trained the target NNs only on the data of individual companies, which is a sufficient amount of data; this helped avoid problems due to training NNs on a small amount of data. We conducted various types of experiments to empirically show that training NNs on a sufficient amount of data is critical in improving their performance. Different types of NNs trained on the data of individual companies outperformed the target NNs trained on the data of the S&P 500.

Our method is conceptually simple and easy to apply. To the best of our knowledge, no previous works have attempted to predict the future price of a stock index without using the data of the stock index in the training process. Although we tested our method on only basic NNs, it could be easily applied to more sophisticated NNs or other machine learning models, as long as the data of individual companies are available.
